# Early Follicular Phase Human Chorionic Gonadotropin Addition May Improve the Outcomes of *In Vitro* Fertilization/Intracytoplasmic Sperm Injection in Patients With “Unpredictable” Poor Response to Gonadotropin-Releasing Hormone Antagonist Protocol

**DOI:** 10.3389/fendo.2021.739773

**Published:** 2021-10-11

**Authors:** Chunhui Zhang, Fangrong Wu, Zexuan Wu, Bolan Sun, Cheng Chen, Weiping Qian

**Affiliations:** Reproductive Medicine Center, Peking University Shenzhen Hospital, Shenzhen, China

**Keywords:** ovarian stimulation, unpredictable POR, *in vitro* fertilization, low-dose hCG, GnRH antagonist

## Abstract

**Purpose:**

To compare the effects of early and mid-late follicular phase administration of 150 IU of human chorionic gonadotropin (hCG) on gonadotropin-releasing hormone (GnRH) antagonist protocol in “unpredictable” poor ovarian response (POR) women undergoing *in vitro* fertilization/intracytoplasmic sperm injection (IVF/ICSI) treatment.

**Methods:**

A retrospective single-center cohort study was conducted on 67 patients with “unpredictable” POR in their first IVF/ICSI cycle receiving GnRH antagonist protocol. Patients were treated with a second IVF/ICSI cycle using the same GnRH antagonist protocol with the same starting dose of recombinant follicle-stimulating hormone (rFSH) as the first cycle; a daily dose of 150 IU of hCG was administrated on either stimulation day 1 (Group A, n = 35) or day 6 (Group B, n = 32). The number of oocytes retrieved, number of usable embryos, serum level of estradiol (E_2_) on day of hCG trigger, and clinical pregnant outcomes were studied.

**Results:**

The addition of 150 IU of hCG on either the first day or sixth day of stimulation increases the serum level of E_2_, luteinizing hormone (LH), and hCG on the day of hCG trigger. Only the use of 150 IU of hCG on the first stimulation day improved the number of oocytes retrieved, mature of oocytes, and usable embryos, but not the addition of hCG on stimulation day 6. Implantation rate, clinical pregnancy rate, and ongoing pregnancy rate showed an increasing trend in patients receiving 150 IU of hCG in the early phase compared with mid-late phase, even thought there was no statistically significant difference.

**Conclusions:**

Our study demonstrated that adding 150 IU of hCG in subsequent GnRH antagonist cycle in “unpredictable” poor responders is associated with the improvement of response to stimulation. Furthermore, early follicular phase addition of 150 IU of hCG significantly increased the number of oocytes retrieved and usable embryos than did the mid-late addition of the same dose.

## Introduction

A successful pregnancy of *in vitro* fertilization (IVF) treatment depends on the number and quality of oocytes retrieved. Ovarian stimulation (OS) could obtain multiple oocytes in one treatment cycle (1). During OS, gonadotropin-releasing hormone (GnRH) antagonist is used to inhibit the luteinizing hormone (LH) surge to prevent the premature ovulation. Except for the trigger of final maturation of oocyte and ovulation, LH also plays an essential role in the development of oocyte, and oversuppressed LH level might be related to inferior embryo quality and early pregnancy loss (2–4). Therefore, LH supplementation during OS treatment is proposed, even though the threshold of LH for normal follicular development remains controversial. The expert consensus in the Asia-Pacific region suggested that LH supplementation might benefit patients aged ≥35 years with poor or suboptimal response to standard OS protocol (5).

Because of the homology between human chorionic gonadotropin (hCG) and LH, hCG has been widely used as an alternative to endogenous LH (3, 6). Compared with recombinant LH (rLH), hCG possesses a longer half-life and a higher affinity to LH/hCG receptor (6). Serafini et al. reported that addition of low-dose hCG in late follicular phase in patients with low LH level could improve IVF outcomes by generating a high number of top-quality embryos (7). Drakakis et al. demonstrated that daily administration of 100 IU of hCG throughout the early follicular phase improved implantation and clinical pregnant rates in women aged 35–40 years (8). However, in their early studies, they could not obtain any improvement in clinical pregnancy rate by adding rLH in mid-late follicular phase during OS in women aged >35 years (2, 9). Another prospective randomized controlled trial (RCT) reported that compared with a daily injection of 50 and 100 IU, 150 IU of hCG could retrieve a significantly higher number of oocytes (6). Therefore, the dose and time of adding hCG to optimize the development of oocytes during OS are still controversial.

The purpose of this retrospective study was to define a possible optimal dose and timing for the addition of hCG to routine GnRH antagonist protocol in ‘‘unpredictable” poor responding patients.

## Materials and Methods

### Study Population

**Figure 1:**
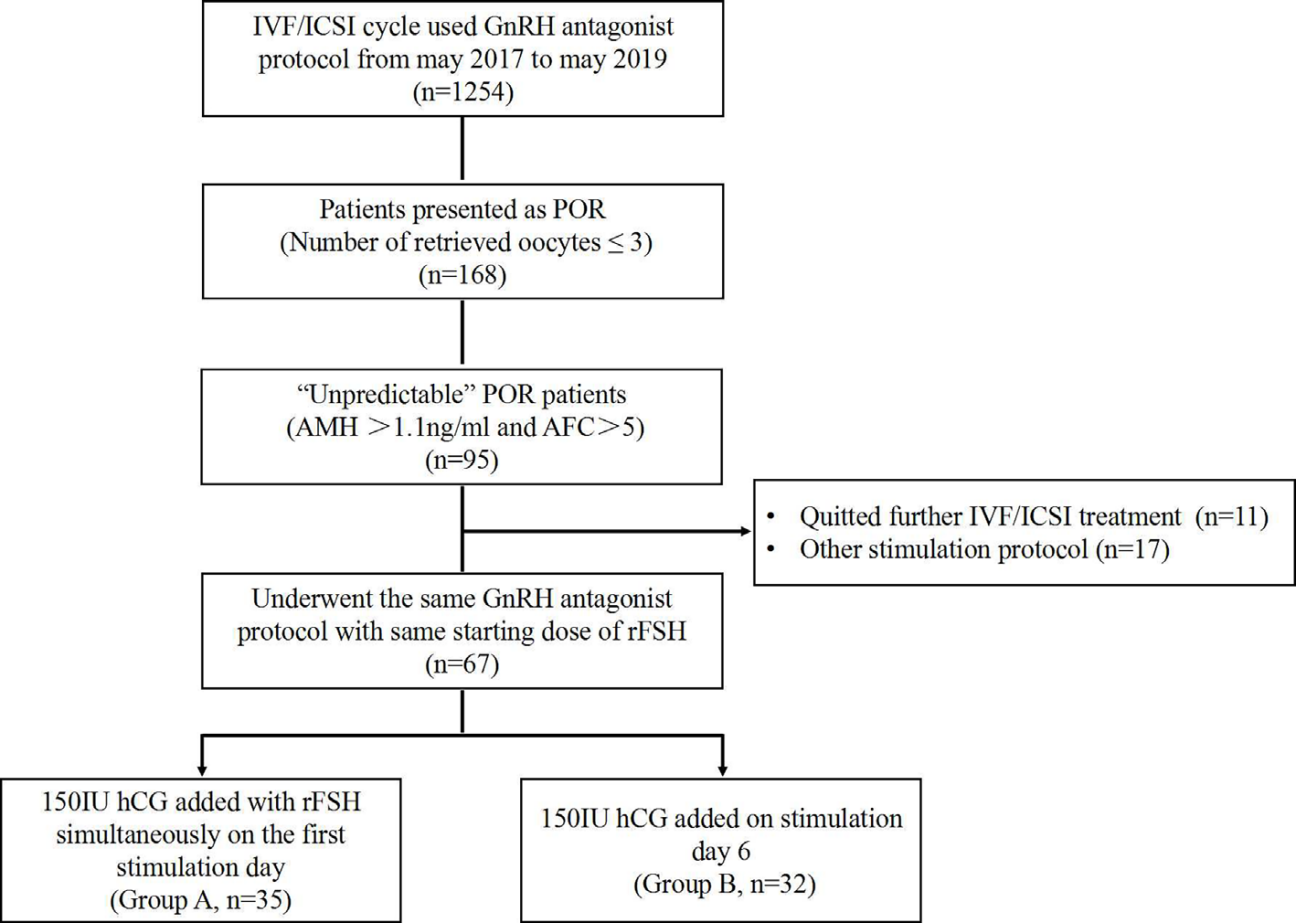
Patient inclusion flow chart.

This retrospective study was conducted at the Reproductive Medicine Center of Peking University Shenzhen Hospital between May 2017 and May 2019. The approval from the institutional review board was obtained at the initiation of the study. Eligible women aged 21–40 years, searching for IVF/intracytoplasmic sperm injection (IVF/ICSI) treatment by using GnRH antagonist stimulation protocol, were analyzed. The inclusion criteria include the following: 1) has a normal ovarian reserve, including anti-Müllerian hormone (AMH) >1.1 ng/ml and antral follicle count (AFC) >5 (10); 2) showed a “unpredictable” poor ovarian response (POR) as ≤3 oocytes were retrieved in the first IVF/ICSI cycle; and 3) used the same GnRH antagonist protocol with hCG addition in the second cycle (**Figure 1**).

### Study Design

In the first IVF/ICSI cycle, all patients used the standard GnRH-antagonist protocol as OS. The starting dose of recombinant follicle-stimulating hormone (FSH) (rFSH; GONAL-f, Merck Serono) for OS was individualized on patient’s ovarian reserve testing (11). A dose of rFSH that ranged from 150 to 300 IU per day was started on the second day of menstruation and was adjusted according to the growth of follicle and estradiol (E_2_) level. A daily dose of 0.25 mg of GnRH antagonist (Cetrotide, Merck Serono) was added on stimulation day 6. Final oocyte maturation was triggered using 2,000 IU of hCG and 0.2 mg of triptorelin (Decapeptyl; Ferring GmbH, Germany) when there were ≥2 follicles of ≥18 mm in average diameter measured in two dimensions. On stimulation day 6 and hCG administration for final oocyte maturation, serum sex hormone levels were measured. The minimum detectable dose (MDD) was 0.1 mIU/ml for FSH, 0.05 mIU/ml for LH, and 0.16 mIU/ml for hCG. All serum tests were drawn in the morning before any treatments were administered. Oocyte retrieval was performed 36 h later. Zhang and Qian, who have more than 10 years’ experiences in ovum pick-up operation, specifically designed the procedure. Insemination methods were chosen depending on semen parameters. Embryological procedures followed the standard protocols. All of the patients’ embryo transfer (ET) was canceled because of diminished number of available embryos.

The second IVF/ICSI cycle was performed 3 months later after the first cycle. During this interval, no medications, such as dehydroepiandrosterone (DHEA), coenzyme Q10 (CoQ10), or vitamin D, were described. Patients started the OS with the same rFSH dose, and GnRH antagonist was added on stimulation day 6. During the OS, a daily dose of hCG (150 IU) was administrated and maintained until the day of hCG trigger. According to the start time of hCG administration, all patients were classified into two groups: Group A, hCG started on stimulation day 1; and Group B, hCG added on stimulation day 6. Final oocyte maturation trigger, oocyte retrieval, insemination, and embryological procedures were conducted as the first cycle. A maximum of two embryos were transferred 3 days after oocyte retrieval. Luteal phase support consisted of progesterone vaginal suppositories 200 mg three times daily beginning on the evening of oocyte retrieval and continuing until 10 weeks’ estimated gestational age. Clinical pregnancy was confirmed by ultrasonographic visualization of gestational sacs at 4–5 weeks after ET, which includes ectopic pregnancy. Miscarriage was defined as spontaneous clinical pregnancy losses before the 20 completed weeks of gestational age. Implantation rates was calculated as the number of gestational sacs divided by the number of embryos transferred. Ongoing pregnancy was defined as a viable pregnancy that lasted at least at 20 weeks of gestational age (12).

### Statistical Analysis

All analyses were performed using SPSS 22 for Windows. Exploratory data analysis was initially performed to determine the normality of the data. The parametric continuous variables were analyzed by using Student’s t-test, and the results are expressed as mean standard deviation. Percentages or rates were compared by using either chi-square or Fisher’s exact test, as indicated. For comparison of quantitative variables with normal distribution between the four cycles and the two groups, one-way ANOVA was applied. *P*-value <0.05 was considered as statistically significant.

## Results

Sixty-seven patients aged from 23 to 40 years who underwent two cycles of OS and IVF/ICSI were recruited (**Figure 1**). Thirty-five women who received the first hCG (150 IU) injection concomitantly with rFSH on stimulation day 1 were classified as Group A; 32 women who received the first injection of hCG on stimulation day 6 were grouped as Group B. No statistically significant differences were observed in female age, body mass index (BMI), duration of infertility, basal sex hormone level, AMH, AFC, starting dose of rFSH, and IVF/ICSI indication between the two groups (**Table 1**).

**Table 1:** Comparison of baseline characteristics of patients in group A and group B.

	Group A (n = 35)	Group B (n = 32)	95% CI of the different limits		*p*-Value
			Lower	Upper	
Age (years)	30.5 ± 4.6	31.3 ± 3.8	−2.9	1.2	0.163
BMI (kg/m^2^)	22.9 ± 1.8	22.5 ± 1.7	−0.5	1.3	0.981
Infertility duration (years)	3.2 ± 1.3	3.3 ± 1.1	−0.7	0.5	0.431
Basal FSH (mIU/ml)	6.1 ± 2.1	6.8 ± 1.7	−1.5	0.3	0.186
Basal LH (mIU/ml)	7.3 ± 2.4	6.7 ± 2.1	−0.5	1.8	0.421
Basal E_2_ (pg/ml)	39.9 ± 12.7	38.6 ± 10.5	−4.4	7.1	0.216
AMH (ng/ml)	2.7 ± 0.9	2.7 ± 0.7	−0.5	0.3	0.272
AFC (n)	5.7 ± 1.3	5.9 ± 1.2	−0.8	0.5	0.391
Starting dose of rFSH (IU)	225 ± 63	227 ± 62	−32.8	28.1	0.864
IVF/ICSI indication (n/%)					
Tubal factor	15/43%	14/44%	0.4	2.7	0.941
Endometriosis	10/29%	8/25%	0.4	2.5	0.742
Male factors	8/23%	7/22%	0.3	3.0	0.923
Unexplained infertility	2/6%	3/9%	0.3	10.9	0.569

Values are presented as mean ± SD or number (percentage); independent-sample t-test and chi-square test were applied accordingly.

BMI, body mass index; FSH, follicle-stimulating hormone; LH, luteinizing hormone; AMH, anti-Müllerian hormone; AFC, antral follicle count; rFSH, recombinant follicle-stimulating hormone; IVF, in vitro fertilization; ICSI, intracytoplasmic sperm injection.

OS results were compared between the first and second IVF/ICSI cycles (**Tables 2**, **3**). After the supplement of hCG on different days of OS during the second cycle, eight patients in Group A and 21 patients in Group B still showed POR, as ≤3 oocytes were retrieved. ET in nine patients in Group A and seven patients in Group B was canceled because of diminished number of available embryos in the second IVF/ICSI treatment cycle.

**Table 2:** Self-comparison of OS characteristics in group A (n = 35) with and without hCG addition.

	First cycle (rFSH only)	Second cycle (rFSH+hCG on day 1)	95% CI of the different limits		*p*-Value
			Lower	Upper	
E_2_ on stimulation day 6 (pg/ml)	77.5 ± 31.8	75.5 ± 43.9	−16.2	20.4	0.211
Hormone levels on day of hCG trigger					
E_2_ (pg/ml)^*^	378 ± 111.7	1,131.3 ± 444.4	−907.8	−598.7	<0.001
LH (mIU/ml)^*^	1.2 ± 0.7	2.8 ± 1.1	−2.1	−1.2	0.034
hCG (mIU/ml)^*^	0.4 ± 0.2	6.1 ± 2.4	−6.5	−4.9	<0.001
P (pg/ml)	0.6 ± 0.3	0.7 ± 0.3	−0.2	0.08	0.547
Total rFSH used (IU)	2,061.4 ± 670.2	2,106.4 ± 716.7	−375.9	285.9	0.495
Days of stimulation (n)	9.2 ± 1.5	9.3 ± 1.6	−0.9	0.6	0.740
No. of ≥14-mm follicles (n)*	3.3 ± 1.4	8.1 ± 3.2	−5.9	−3.6	<0.001
No. of retrieved oocytes (n)*	1.9 ± 1.4	6.1 ± 3.4	−5.4	−3.0	<0.001
Retrieved oocytes rate (%)^*^	57% (66/115)	76% (214/283)	1.6	4.1	<0.001
No. of mature oocytes (n)^a,^*	1.4 ± 1.1	4.1 ± 2.5	−3.6	−1.8	<0.001
No. of embryos (n)*	0.9 ± 0.9	2.5 ± 1.4	−2.2	−1.1	0.015

Values are presented as mean ± SD or number (percentage); independent-sample t-test and chi-square test were applied accordingly.

OS, ovarian stimulation; hCG, human chorionic gonadotropin; rFSH, recombinant follicle-stimulating hormone; LH, luteinizing hormone; ICSI, intracytoplasmic sperm injection.

a No. of mature oocytes compared only in 14 ICSI cycles.

^*^p-Value < 0.05.

**Table 3:** Self-comparison of OS characteristics in Group B (n = 32) with and without hCG addition.

	First cycle (rFSH only)	Second cycle (rFSH+hCG on day 1)	95% CI of the different limits		*p*-Value
			Lower	Upper	
E_2_ on stimulation day 6 (pg/ml)	72.7 ± 30.8	73.6 ± 31.5	−16.5	14.6	0.684
Hormone levels on day of hCG trigger					
E_2_ (pg/ml)^*^	410.2 ± 198.9	946.3 ± 337.6	−674.5	−397.6	<0.001
LH (mIU/ml)^*^	1.2 ± 0.7	2.5 ± 1.1	−1.8	−0.9	0.005
hCG (mIU/ml)^*^	0.4 ± 0.2	6.4 ± 2.5	−6.8	−5.0	<0.001
P (pg/ml)	0.6 ± 0.3	0.7 ± 0.3	−0.3	0.5	0.150
Total rFSH used (IU)	2,001.6 ± 560.7	2,121.1 ± 733.2	−455.7	206.6	0.120
Days of stimulation (n)	9.0 ± 1.3	9.3 ± 1.5	−0.9	0.5	0.272
No. of ≥14-mm follicles (n)	3.9 ± 1.0	4.9 ± 1.2	−1.6	−0.4	0.462
No. of retrieved oocytes (n)	1.9 ± 1.3	3.3 ± 1.5	−2.1	−0.6	0.854
Retrieved oocytes rate (%)	58% (62/106)	59% (119/202)	0.6	1.6	0.943
No. of mature oocytes (n)^a^	1.3 ± 1.0	3.0 ± 1.6	−2.4	−1.0	0.080
No. of embryos (n)	1.0 ± 0.9	1.8 ± 0.8	−1.2	−0.3	0.760

Values are presented as mean ± SD or number (percentage); independent-sample t-test and chi-square test were applied accordingly.

OS, ovarian stimulation; hCG, human chorionic gonadotropin; rFSH, recombinant follicle-stimulating hormone; LH, luteinizing hormone; ICSI, intracytoplasmic sperm injection.

a No. of mature oocytes compared only in 12 ICSI cycles.

^*^p-Value < 0.05.

In Group A, the daily addition of hCG (150 IU) at the beginning of stimulation significantly improved the total number of ≥14-mm follicles (8.1 ± 3.2 *vs.* 3.3 ± 1.4) and oocytes retrieved (6.1 ± 3.4 *vs.* 1.9 ± 1.4) compared with that at the first cycle. Accordingly, retrieved oocyte rate (76% *vs.* 57%) and the number of mature oocytes in ICSI cycle (4.1 ± 2.5 *vs.* 1.4 ± 1.1) were also significantly increased after the hCG addition. Mean serum E_2_ (1,131.3 ± 444.4 *vs.* 378.1 ± 111.7), LH (2.8 ± 1.1 *vs.* 1.2 ± 0.7), and hCG (6.1 ± 3.4 *vs.* 1.9 ± 1.4) level on the day of hCG trigger were significantly higher in the second cycle compared with the first. There was no significant difference in E_2_ level on stimulation day 6, progesterone concentration on the day of hCG trigger, total dosage of rFSH used, and days of stimulation between the two cycles (**Table 2**).

Details referring to OS and embryological results of Group B are summarized in **Table 3**. The same as Group A, Group B showed a higher serum level of E_2_ (946.3 ± 337.6 *vs.* 410.2 ± 198.9), LH (2.5 ± 1.1 *vs.* 1.2 ± 0.7), and hCG (6.4 ± 2.5 *vs.* 0.4 ± 0.2) on hCG trigger day compared with the first cycle. But the number of ≥14-mm follicles, oocytes retrieved, mature oocytes in ICSI cycle, and retrieved oocyte rate showed no difference in Group B as hCG added on the stimulation day 6 compared without it. E_2_ level on stimulation day 6, progesterone concentration on the day of hCG trigger, total dosage of rFSH used, and days of stimulation also showed no differences between two cycles.

**Table 4** compares the outcomes of the second cycle between Groups A and B. E_2_ level on day of hCG trigger (1,131.3 ± 444.4 *vs.* 946.3 ± 337.6), number of oocytes retrieved (6.1 ± 3.4 *vs.* 4.9 ± 2.3), and number of available embryos (2.5 ± 1.4 *vs.* 1.8 ± 0.8) were significantly higher in patients receiving 150 IU of hCG on the first day of stimulation (Group A) than on stimulation day 6 (Group B). Implantation rate, clinical pregnancy rate, and live birth rate showed an increasing trend in Group A than in Group B, with no statistically significant difference.

**Table 4:** Comparison of IVF/ICSI outcomes between group A and group B.

	Group A (n = 35)	Group B (n = 32)	95% CI of the different limits		*p*-Value
			Lower	Upper	
E_2_ on day of hCG trigger (pg/ml)^*^	1,131.3 ± 444.4	946.3 ± 337.6	−8.9	378.9	0.043
Stimulation duration (days)	9.3 ± 1.6	9.4 ± 1.7	−0.9	0.7	0.454
hCG duration (days)	9.3 ± 1.6	3.3 ± 1.4	5.4	6.8	0.650
No. of retrieved oocytes (n)*	6.1 ± 3.4	4.9 ± 2.3	−0.2	2.6	0.006
No. of embryos (n)*	2.5 ± 1.4	1.8 ± 0.8	0.2	1.4	0.010
Endometrial thickness on day of hCG trigger (mm)	9.7 ± 2.0	9.8 ± 1.8	−1.1	0.8	0.227
No. of embryos transferred (n)	1.5 ± 0.7	1.3 ± 0.8	−0.2	0.5	0.169
**Pregnant outcomes (%/n)**					
Implantation	20% (11/60)	13% (6/46)	0.23	2.0	0.462
Clinic pregnancy rate/transfer	29% (9/31)	24% (6/25)	0.20	2.6	0.672
Miscarriage	18% (2/9)	33% (2/6)	−0.5	0.4	0.804
Ongoing pregnancy	64% (7/9)	50% (3/6)	−0.9	0.9	0.224
Live birth	64% (7/9)	50% (3/6)	−0.9	0.9	0.224

Values are presented as mean ± SD or number (percentage); independent-sample t-test and chi-square test were applied accordingly.

IVF, in vitro fertilization; ICSI, intracytoplasmic sperm injection; hCG, human chorionic gonadotropin.

^*^p-Value < 0.05.

**Figure 2:**
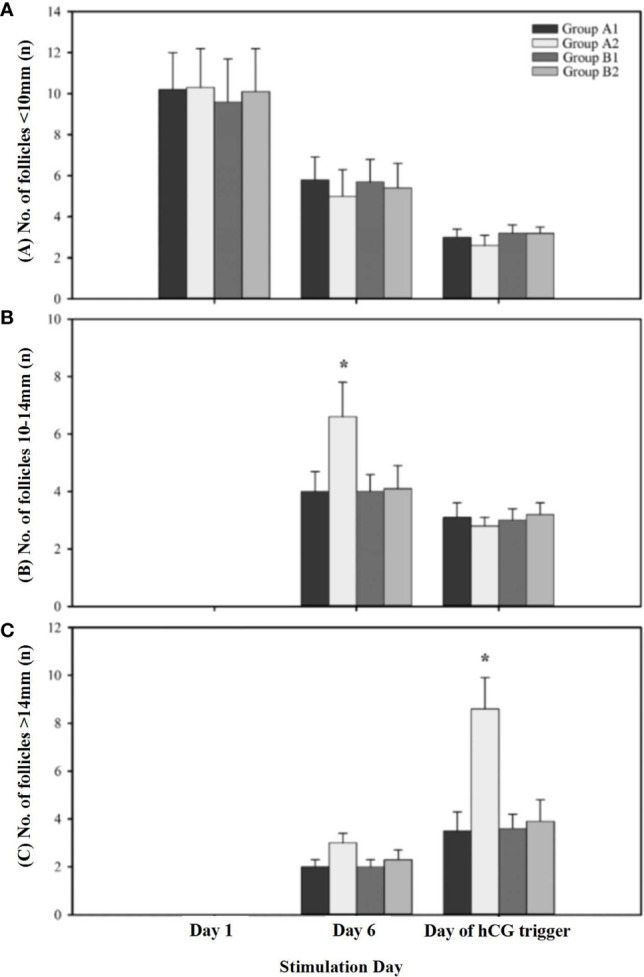
The constitution of different diameters of follicles during ovarian stimulation. **(A)** Presents the number of follicles < 10mm on different stimulation day; **(B)** Presents the number of follicles 10-14mm on different stimulation day; **(C)** Presents the number of follicles > 14mm on different stimulation day. A1 indicates first cycle of Group A; A2 indicates second cycle of Group A; B1 indicates first cycle of Group B; B2 indicates second cycle of Group B. * indicates significant differences (P < 0.05) within treatment groups on the same stimulation day.

The constitution of different-diameter follicles during OS on different stimulation days are shown in **Figure 2**. The number of large follicles (diameter >14 mm) on the day of hCG administration and the number of the medium follicles (diameter 10–14 mm) on stimulation day 6 were significantly higher in the patients receiving 150 IU of hCG on first stimulation day than in other groups. The number of small-diameter follicles (diameter < 10 mm) throughout the whole OS showed no difference among the four groups.

## Discussion

POR generally resulted from diminished ovarian reserve. However, in some cases, poor ovarian responders may present as normal ovarian reserve with AFC >5 and/or AMH >1.1 ng/ml (13, 14). In this retrospective analysis, we evaluated the effects of low-dose hCG supplementation on women with normal AFC and AMH levels but showed a POR to rFSH stimulation in their first cycle. Our results showed that the addition of 150 IU of hCG in those “unpredictable” POR patients could improve their response to OS. For the first time, we compared the effects of different timing of hCG administration on OS outcome. Our results suggested that the addition of 150 IU of hCG in early follicle phase could provide a better clinical outcome compared with that in mid-late phase addition.

Our results were in line with previous studies that the addition of LH/hCG could increase oocyte number and E_2_ production (15, 16). A meta-analysis confirmed that the use of rLH/hCG increased the peak E_2_ levels and oocyte number and improved embryo quality and, therefore, enhanced implantation rate (17). Enrico et al. found that unexpected poor ovarian responders could benefit from addition of 150 IU rLH in subsequent cycle (14). Drakakis et al. demonstrated a better clinical outcome of early hCG administration during OS in women with previous IVF failure compared with rLH (8).

Without LH, FSH alone is inadequate to induce normal follicular development (18). In general, approximately 1% of activated LH receptors are sufficient to maintain follicular development and steroid production. There were also studies that reported that adding rLH/hCG to OS may reduce the number of developing follicles and retrieved oocytes. They explained that a high level of LH activity restricted the development of small ovarian follicles (<10 mm) and inhibited follicular recruitment in patients with normal ovary response (19, 20). Our data showed that the number of medium follicles (10–14 mm) on stimulation day 6 was significantly higher in “unpredictable” POR patients who received 150 IU of hCG at the beginning of rFSH administration than other groups. The reason behind this improvement is that LH binds to the receptor on the theca cells, which increases androgen secretion (21). Androgen improves granulose cell function and increases the size of the follicle of the cohort recruited (22, 23). Therefore, patients with poor response that resulted from deficiency of LH receptor activity may benefit from LH supplementation (24).

Follicular recruitment occurs in the early follicular phase during OS (25). The size of this follicular cohort determines the number of retrieved oocytes in accordance with the number of useable embryos. We found that a higher number of retrieved oocytes and useable embryos were seen in “unpredictable” poor responders who employed 150 IU of hCG in early follicular phase during the second GnRH antagonist stimulation protocol. But the same dose of hCG addition in mid-late follicular phase was not able to increase the number of oocytes and embryos. Therefore, different timing for hCG addition could be a possible reason to explain different conclusions drawn by previous studies. Based on our findings, we proposed the early follicular phase of hCG addition during OS.

In addition, on the day of hCG trigger, endometrial thickness and the progesterone level showed no significant differences between the hCG addition and no additional cycle. Furthermore, the timing of hCG administration, on either the first day or sixth day of OS, also had no effects on endometrial receptivity. The similar implantation and clinical pregnant rate between Group A and Group B in this study have proved it.

## Conclusions

In conclusion, this study firstly showed that early follicular phase addition of hCG significantly increased the number of retrieved oocytes and embryos in patients with “unpredictable” poor response to GnRH antagonist regimens. As a result, the implantation rate, clinical pregnancy rate, and ongoing pregnancy rate were also observed at an increased trend in the “unpredictable” POR patients receiving early hCG supplement. Potential disadvantages of this study include retrospective design and relatively small sample size. There remains a lack of well-designed, randomized, controlled trials evaluating this protocol, and additional research is warranted.

## Data Availability Statement

The original contributions presented in the study are included in the article/supplementary material. Further inquiries can be directed to the corresponding author.

## Ethics Statement

This study was approved by Peking University Shenzhen Hospital Ethics Committee. Written informed consent for participation was not required for this study in accordance with the national legislation and the institutional requirements.

## Author Contributions

CZ and WQ designed the study and performed all ovum pick-up operation; CZ, WQ, BS, and CC performed the ovarian stimulation; CZ, FW and ZW collected and analyzed the data, also wrote the manuscript. All authors contributed and approved the submitted version.

## Funding

This study received funding from National Key Research and Development Program (2018YFC1002104).

## Conflict of Interest

The authors declare that the research was conducted in the absence of any commercial or financial relationships that could be construed as a potential conflict of interest.

The reviewer QX declared a shared affiliation, with no collaboration, with the authors to the handling editor at the time of review.

## Publisher’s Note

All claims expressed in this article are solely those of the authors and do not necessarily represent those of their affiliated organizations, or those of the publisher, the editors and the reviewers. Any product that may be evaluated in this article, or claim that may be made by its manufacturer, is not guaranteed or endorsed by the publisher.
